# Slaughterhouse Wastewater as a Reservoir of Thermotolerant *E. coli* With Antimicrobial Resistance and Virulence Potential in Dhaka, Bangladesh

**DOI:** 10.1155/ijm/2875935

**Published:** 2025-12-06

**Authors:** Nahida Sarwer Chowdhury, Rifah Tasnia, Najmun Nahar, Zenat Zebin Hossain, Jannatul Ferdous, Humaira Akhter, Anowara Begum

**Affiliations:** ^1^ Department of Microbiology, University of Dhaka, Dhaka, Bangladesh, du.ac.bd; ^2^ Department of Public Health, Independent University, Bangladesh, Dhaka, Bangladesh, iub.edu.bd; ^3^ Department of Life Sciences, Independent University, Bangladesh, Dhaka, Bangladesh, iub.edu.bd

**Keywords:** antimicrobial resistance, biofilm, *Escherichia coli*, integrons, plasmid, slaughterhouse

## Abstract

Slaughterhouses are aimed at controlling organic matter and pathogens during animal processing; however, wastewater discharge often introduces microorganisms into the environment. This investigation focused on the isolation and characterization of thermotolerant *Escherichia coli* strains exhibiting pathogenicity, multidrug resistance, and biofilm‐forming capacity from wastewater collected at the Kaptan Bazar slaughterhouse in Dhaka, Bangladesh. Seventy *E. coli* isolates were identified using selective culture media (MacConkey and eosin methylene blue agar) and PCR targeting the *uidA* gene. Antibiotic susceptibility was assessed using the Kirby–Bauer and modified Hodge methods. Biofilm formation was evaluated through the crystal violet assay. The presence of antibiotic resistance, virulence, and biofilm‐associated genes was determined by conventional PCR. The most common virotypes were EI*EC* (7.14%), followed by ET*EC* (2.86%) and EH*EC* (1.43%). Extended‐spectrum *β*‐lactamase (ESBL) genes *blaTEM* (6.94%) and *blaCTX-M-15* (2.78%) were detected. Carbapenem resistance genes included *blaIMP-1* (3.70%), *blaIMP-4* (1.85%), *blaOXA-48* (21.76%), *blaOXA-47* (0.46%), and *blaOXA-1* (1.39%). Eleven isolates tested positive for carbapenemase production via the modified Hodge test. Non‐*β*‐lactam resistance genes detected included *dfrA17* (25.46%), *tetA* (13.89%), *sul2* (6.48%), *qnrS* (6.48%), and *qnrB* (3.24%). Class 1 integrons were present in 16 strains (22.86%), while both Class 2 and 3 integrons were absent. Colistin MIC values ranged from ≤ 0.5 to 2 *μ*g/mL. Plasmid analysis showed that 59 isolates (84.29%) carried plasmids ranging in size from > 500 bp to > 10 kb. The crystal violet assay indicated that 74.29% of the isolates were biofilm producers, with 68.57% forming weak biofilms. Most weak biofilm formers and all moderate biofilm formers carried multiple antibiotic resistance genes. The results underscore a significant presence of antimicrobial‐resistant and biofilm‐producing *E. coli* in slaughterhouse effluents, highlighting the potential dissemination of ARGs into the surrounding ecosystem and food chains, posing a serious public health risk. The evidence also points to the urgent necessity for enhanced hygiene and treatment protocols to mitigate environmental and public health risks.

## 1. Introduction

Slaughterhouses generate significant quantities of wastewater during animal processing, cleaning operations, and equipment sanitation [1]. This waste is a potential source of diverse pathogens such as bacteria, viruses, prions, and parasites. It is often repurposed in animal feed production, contributing to the ongoing cycle of exposure. These supplements can be added to animal feed or used on their own. Despite this, large amounts of slaughterhouse waste are generated around the world, making its disposal a huge logistical challenge for meat and poultry processing industries. Due to the high volume of animal waste and rising treatment costs, improper and unsafe disposal remains a significant concern. As a result of such practices, major environmental issues may arise [2, 3].

*Escherichia coli*, a natural inhabitant of the gastrointestinal tracts of humans and animals, can enter environmental systems through fecal discharge or wastewater. It serves as a standard indicator of fecal pollution in a variety of places, including wastewater and feed. This indicator organism also contributes to the global problem of antibiotic resistance [4]. Slaughterhouses have been implicated in the spread of multidrug‐resistant *E. coli* and the transmission of resistant bacteria, as well as their transportable genetic components throughout the food web [5]. For example, a recent study in India reported that over one‐quarter of *E. coli* isolates from pig slaughterhouse effluents were extended‐spectrum beta‐lactamase (ESBL) producers, including strains carrying the *bla*NDM‐1 carbapenemase gene [6]. Recognizing the molecular mechanisms by which resistance genes are acquired or transmitted is crucial for developing new antimicrobial strategies and preventive measures to limit the spread of resistance determinants among pathogens. The spread of antibiotic resistance in bacteria is largely driven by genetic mutations and the horizontal exchange of mobile elements (plasmids, transposons, and bacteriophages), which together accelerate the rapid dissemination of resistance genes.

Antibiotic resistance genes′ development and spread is a severe problem in the treatment of infectious zoonotic diseases. Two genetic bacterial systems, chromosomal DNA and extrachromosomal DNA, play a role in gene transmission through a variety of processes involving mutations, recombination, and horizontal gene transfer, resulting in bacterial genomic diversity. As a result, MDR plasmids aid in the propagation of resistance through horizontal gene transfer.

In general, *E. coli* strains have a higher fraction of genomic plasmid codes responsible for antibiotic resistance than those of the chromosome [4]. Extended‐spectrum beta‐lactam antibiotics are one of the most valuable treatments in therapeutic applications, and ESBL promotes resistance to them. ESBL‐producing *E. coli* found in slaughterhouses poses an extreme threat to human health [7]. Precautionary measures and hygienic environments are major needs to address and get over. This pathogenic organism, which was isolated from surfaces in contact with foods in a poultry slaughterhouse cutting room, has been reported to produce biofilm, which could lead to the persistence of this organism during food processing, increasing the risk of food contamination and endangering the health of consumers [8, 9].

Enterohemorrhagic *E*. *coli* (EH*EC*) is a kind of gastrointestinal pathogenic *E. coli* that causes significant clinical signs such as hemorrhagic colitis and the potentially fatal hemolytic uremic syndrome. Household ruminants, such as cattle, sheep, and goats, have been identified as EH*EC* strains′ principal natural hosts, making them zoonotic diseases. Several outbreaks of *E. coli* O157:H7 have been associated with both recreational and drinking water sources, underscoring the critical importance of effectively removing these pathogens from human and animal waste [10, 11].

So far, no data on the occurrence, phenotypic, and genotypic properties of ESBL‐producing *E. coli* in wastewater from slaughterhouses in Bangladesh have been published. However, recent studies in neighboring South Asian countries have reported a significant prevalence of antibiotic‐resistant *E. coli* in slaughterhouse effluents—for instance, in India [6] and via One Health surveillance in Nepal [12]—highlighting this issue as a regional concern. This study is therefore the first in Bangladesh to isolate and characterize antibiotic‐resistant, biofilm‐forming *E. coli* strains from slaughterhouse wastewater, addressing an important knowledge gap in our understanding of environmental AMR reservoirs in this context.

## 2. Materials and Methods

### 2.1. Study Area

Wastewater samples were obtained from the Kaptan Bazar Slaughterhouse located in Dhaka, Bangladesh (coordinates: N23° 41.004″, E90° 27.080″; elevation: 7 m). Sampling was conducted monthly between September 2019 and March 2020, before any treatment stage, as no wastewater treatment facility was operational at the site. At each time point, one representative grab sample (2 L) was collected in the morning (approx. 10:00 a.m.). From each grab, 500 mL was aliquoted into sterile 1‐L containers, transported to the laboratory at the University of Dhaka in a cool box within 2 h, and processed on the same day.

### 2.2. Isolation of *E. coli* Strains

To isolate *E. coli*, 100 *μ*L of each wastewater aliquot was plated on modified thermotolerant agar (m‐TEC, Oxoid, Basingstoke, United Kingdom) and incubated at 45°C for 24 h. Colonies exhibiting a purple color were selected and further cultured on EMB (eosin methylene blue) (Oxoid, Basingstoke, United Kingdom) agar and incubated overnight at 37°C. Green metallic sheen colonies were subsequently streaked onto MacConkey agar and incubated for 24 h at 37°C, followed by streaking onto nutrient agar for overnight incubation at 37°C. From each sample, approximately 10–12 presumptive colonies were picked, of which 8–10 were confirmed as thermotolerant *E. coli* using conventional and molecular techniques.

### 2.3. Polymerase Chain Reaction (PCR) Procedures

#### 2.3.1. DNA Extraction and General PCR Conditions

DNA templates for PCR were prepared using the boiling method: Briefly, bacterial colonies were suspended in 100 *μ*L of sterile nuclease‐free water, heated at 100°C in a heat block for 10 min, immediately cooled on ice for 10 min, and centrifuged at 12,000 rpm for 5 min; the resulting supernatant containing crude DNA was used as the template. The same DNA preparations were used for all subsequent PCR analyses except for plasmid profiling. PCR assays were conducted using a PTC‐100 thermal cycler (MJ Research, United States) following general parameters unless noted otherwise: an initial denaturation at 95°C for 3 min, followed by 35 cycles of denaturation at 95°C for 45 s, annealing at the specified temperature, and extension at 72°C for 45 s, with a final extension at 72°C for 7 min. Each 25 *μ*L reaction mixture contained 10X PCR buffer, 200 *μ*M of each dNTP, 400 nM of each primer, 2–5 U Taq DNA polymerase (Takara Ex Taq, Japan), and 250 ng of template DNA. Sterile distilled water was added to adjust the reaction volume, and negative controls (no template DNA) were included for each run. (Details of primer and target gene information for PCR are provided in Table S1.)

#### 2.3.2. Identification of *E. coli* Strains

PCR was conducted to detect the conserved *uidA* gene using the standard protocol described above. The annealing temperature for this reaction was 55.2°C. Amplified products were separated by electrophoresis on 1.5% agarose gels, stained with GoldView I (Zomanbio Co., Beijing, China), and visualized under UV light.

#### 2.3.3. Detection of Virulence and Antibiotic Resistance Genes

Virulence and antibiotic resistance genes were amplified using gene‐specific primers listed in the supporting table. PCR reaction mixtures and cycling conditions followed the general protocol, with slight modifications:

• The initial denaturation was at 94°C for 5 min.
• The annealing temperatures varied as detailed in the supporting table.
• A final extension was performed at 72°C for 5 min.

PCR products were analyzed via gel electrophoresis on 1.5% agarose gels stained with ethidium bromide and visualized using a Gel Doc 2000 documentation system (Bio‐Rad, Hercules, California, United States).

#### 2.3.4. Detection of Integrons

The presence of Class 1, Class 2, and Class 3 integrons was validated by targeting the respective *intI1*, *intI2*, and *intI3* genes. DNA templates were prepared using the boiling method [13]. Reaction mixtures were prepared as described, with annealing temperatures set at 60°C for *intI1* and 55°C for *intI2* and *intI3*. Amplification conditions included 35 cycles with an extension of 1 min at 72°C for *intI2* and *intI3* and 30 s for *intI1*.

### 2.4. Extraction Plasmid DNA

According to the manufacturer′s instructions, plasmid DNA was extracted using the GeneJET Plasmid Miniprep Kit (Thermo Scientific, Waltham, Massachusetts, United States).

### 2.5. Carbapenemase Activity Detection

Carbapenemase activity was evaluated via the modified Hodge test (MHT), utilizing Mueller–Hinton agar (MHA) and carbapenem disks (imipenem [IMP] and meropenem [MEM]) in accordance with Clinical and Laboratory Standards Institute (CLSI) recommendations (CLSI, 2014; CLSI M100‐S26). Control strains included *E. coli* ATCC 25922, *Klebsiella pneumoniae* ATCC 1706, and *Pseudomonas aeruginosa* ATCC 27853.

### 2.6. Minimum Inhibitory Concentration (MIC) Determination

MICs for colistin were determined in cation‐adjusted Mueller–Hinton II broth using colistin sulfate powder (Sigma‐Aldrich Inc.), following the broth microdilution methodology recommended by the CLSI guideline (CLSI, 2014; M100‐S26). After incubation for 16–20 h at 35°C, the microdilution panels were analyzed, and MIC values were recorded. Since CLSI 2014 did not provide specific breakpoints for colistin in Enterobacteriaceae, interpretation was made using the criteria available at that time. We note that subsequent CLSI documents (M07, M100, and MR01, 2020) have since updated methodological and interpretive guidance for colistin.

### 2.7. Biofilm Formation Assay

Biofilm formation was evaluated using a modified protocol based on the method described by O′Toole and Kolter (1998). In this experiment, *E. coli* isolate biofilm formation was quantified using the crystal violet staining method in 96‐well plates with TSB medium. Optical density (OD570) readings determined the strength of biofilm production. The staining procedure and the extent of biofilm formation were determined as described previously by Schiebel et al. [14].

### 2.8. Identification of Biofilm‐Associated Genes

PCR evaluated biofilm‐associated genes for the genes named *fimH*, *fliC*, *csgA*, *papC*, and *pap*G. The supporting table shows primer sets (BioTeZ Berlin‐Buch GmbH, Berlin, Germany) and sizes of PCR products. The boiling method described in Section 2.4 was used to obtain DNA templates. The thermal cycling conditions were completed following the protocol according to Schiebel et al. [14].

### 2.9. Detection of Cellulose and Curli Fimbriae

In *E. coli*, curli fibers and cellulose expression assist biofilm formation [15]. Congo red dye can be used to examine the construction of these matrix components, which bind to both cellulose and curli [14]. The production of cellulose and curli fimbriae was determined by streaking *E. coli* isolates on LB agar plates without salt supplemented with 40 *μ*g/mL Congo red (Carl Roth GmbH, Karlsruhe, Germany) and 20 *μ*g/mL Brilliant Blue R‐250 (Carl Roth GmbH). The morphotypes were identified and classified as red, dry, and rough (rdar) morphotypes designating curli and cellulose production; brown, dry, and rough (bdar) morphotypes targeting only curli production; and smooth and white (saw) morphotypes indicating neither curli nor cellulose production after incubation for 24 h at 37°C and 48 h at 28°C [14].

## 3. Results

### 3.1. Isolation and Identification of *E. coli* Isolates

Over a 7‐month period, 70 *E. coli* isolates were successfully recovered and identified through a combination of traditional microbiological and molecular methods. PCR analysis identified virulence‐associated genes among the isolates. Two isolates (IS13 and IS14) were confirmed as enterotoxigenic *E. coli* (ET*EC*) based on the presence of *eltB* (322 bp) and *estA* (147 bp), while one isolate (IS110) was identified as EH*EC* due to the detection of *eaeA* (376 bp) and *vt1* (130 bp). The *ipaH* gene (423 bp), indicative of enteroinvasive *E. coli* (EI*EC*), was detected in five isolates (IS4, IS12, IS29, IS38, and IS42). Other virulence genes (*vt2*, *ial*, *pCVD*, and *bfpA*) were absent (the gel figures are provided in Figure S1).

### 3.2. Prevalence of Antibiotic‐Resistant Genes and Integron Association

**Figure 1:**
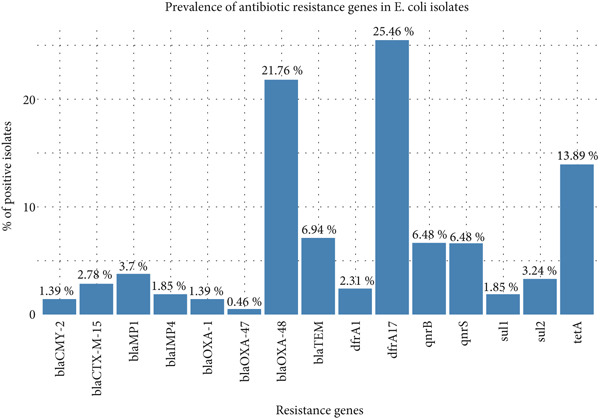
Resistance gene diversity of *E. coli* isolates.

**Figure 2:**
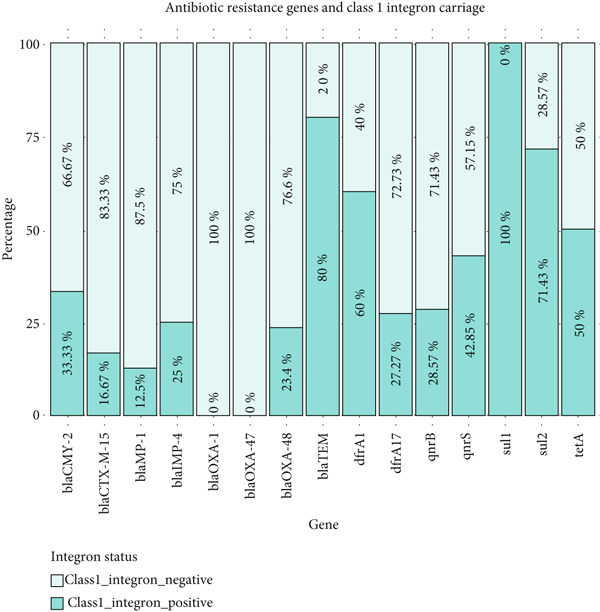
Association of resistance genes and integrons.

The *dfrA17* gene was the most commonly detected resistance determinant, present in 25.46% of the isolates, followed by *blaOXA-48* (21.76%) and *tetA* (13.89%). Other resistance genes were detected at comparatively lower frequencies, ranging from 0.46% (*blaOXA-47*) to 6.94% (*blaTEM*) (Figure 1). None of the isolates carried *blaNDM-1*, *blaKPC1*, *blaKPC2*, *blaKPC3*, *blaVIM1*, *blaVIM2*, *mcr-1*, *mcr-2*, *mcr-3*, *mcr-4*, or *mcr-5*. Screening for integrons revealed that none of the isolates was positive for Class 2 or Class 3 integrons, whereas 16 out of 70 isolates (22.86%) harbored Class 1 integrons, which were frequently associated with multiple resistance genes (Figure 2). In particular, Class 1 integron‐positive isolates harbored a higher proportion of *blaTEM*, *sul1*, *sul2*, and *dfrA1* genes compared to integron‐negative isolates.

Statistical analysis further confirmed significant associations between Class 1 integrons and several AMR genes, including *blaTEM*, *sul1*, *sul2*, *dfrA1*, *qnrS*, and *tetA* (*p* < 0.05). Full statistical outputs, including odds ratios and confidence intervals for all genes, are provided in Table S2.

### 3.3. Phenotypic Detection of Carbapenemase Production

The antibiotic susceptibility of 12 isolates with reduced carbapenem susceptibility was further evaluated on MHA plates using *E. coli* DH5*α* as the carbapenem‐susceptible reference strain. The test isolates were streaked on MHA plates containing IMP and MEM disks. Of the 12 isolates tested, five exhibited resistances to MEM and another five to IMP, as evidenced by markedly reduced or absent inhibition zones around the respective antibiotic disks. The remaining isolate showed intermediate susceptibility.

### 3.4. MIC of Colistin

MICs for colistin among the 70 *E. coli* isolates ranged from ≤ 0.5 to 2 *μ*g/mL. The peak MIC value of 2 *μ*g/mL was recorded in 14.3% (5) of the isolates, 1 *μ*g/mL for 28.6% (13) isolates, and ≤ 0.5 *μ*g/mL for 57.1% (52) isolates, respectively.

### 3.5. Plasmid Profile Analysis

**Figure 3:**
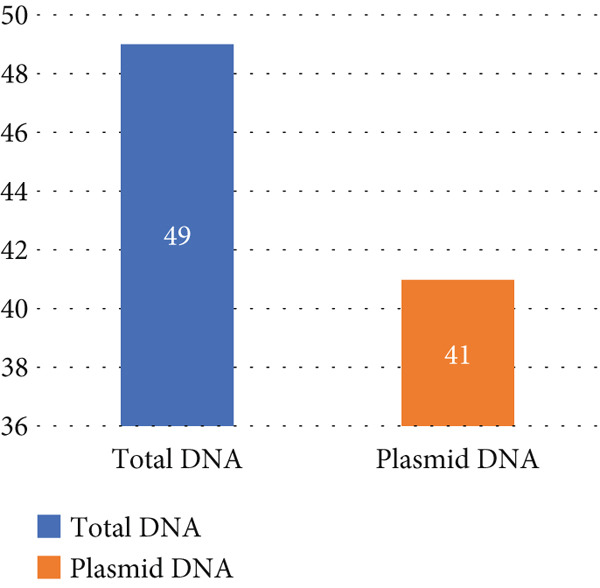
Comparison of harboring resistance genes between total DNA and plasmid DNA.

Plasmid profile analysis of *E. coli* isolates revealed that 59 isolates harbored plasmids of varying sizes (> 500 bp to > 10 kb), with 29 displaying multiple bands and others showing single bands, including shared band sizes among intraspecies isolates. PCR screening for plasmid‐mediated antibiotic resistance genes was conducted on plasmid DNA extracted from 59 isolates, with 41 yielding positive results (Figure 3). All 14 *blaTEM*‐positive isolates in total DNA PCR remained positive in plasmid PCR, while *blaCTX-M-15*, *blaCMY-2*, and *blaOXA-1*, which were detected in total DNA, were not amplified from plasmid DNA, suggesting a chromosomal location for these genes. Among *qnrS*‐positive isolates, 10 were plasmid borne compared to 14 in total DNA, while sul1 was present in both cases. For *sul2*, 13 of 14 total DNA‐positive isolates retained the gene in plasmid PCR, with one being negative (the gel figures are provided in Figure S2).

As plasmid DNA preparations may contain chromosomal DNA contamination, PCR on plasmid extracts cannot conclusively confirm plasmid localization. Therefore, our plasmid PCR results should be considered indicative, and further confirmatory methods would be required to verify true plasmid carriage of these genes.

### 3.6. Biofilm Formation and Presence of Biofilm‐Associated Genes

**Figure 4:**
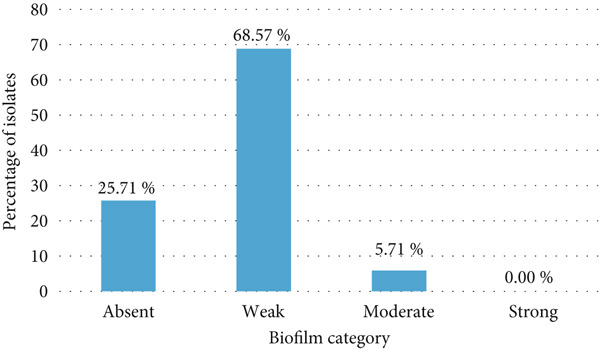
Distribution of bacterial isolates across biofilm categories.

Out of the 70 isolates analyzed, 52 (74.29%) demonstrated biofilm‐forming ability, whereas 18 (25.71%) were nonbiofilm producers. OD at 570 nm readings ranged from 0.047 to 0.161, with 48 (68.57%) being weak and 4 (5.71%) intermediate biofilm producers; no strong biofilm formers were detected (Figure 4). Biofilm‐associated genes varied in prevalence, with *fimH* and *csgA* (100%) being the most common, while *papC* (7.69%) and *papG* (1.92%) were less frequent. No isolate carried the *fliC* gene (Table 1).

**Table 1:** Distribution of biofilm‐associated genes among isolates.

**Biofilm former**	**Cutoff value OD at 570 nm**	**No. of isolates**	**Biofilm-forming genes**				
			***fim*H (** **n** **, %)**	***csg*A (** **n** **, %)**	***fli*C (** **n** **, %)**	***pap*C (** **n** **, %)**	***pap*G (** **n** **, %)**
Strong	0.216 < *x*	0	0	0	0	0	0
Moderate	0.108 < *x* ≤ 0.216	4	4 (100%)	4 (100%)	0	0	1 (25%)
Weak	0.054 < *x* ≤ 0.108	48	48 (100%)	48 (100%)	0	4 (8.33%)	0
Nonbiofilm former	*x* ≤ 0.054	18	18 (100%)	18 (100%)	0	1 (5.55%)	0

### 3.7. Curli and Cellulose Fimbria Production in *E. coli* Isolates

The Congo red agar plates (Table 2) revealed three types of morphotypes after incubation at 28°C and 37°C: rdar (indicating cellulose and curli production), bdar (indicating only curli production), and saw (no cellulose and curli development). There was no isolate that produced the pdar morphotype, which indicates cellulose expression without curli formation.

**Table 2:** Temperature effect on curli production.

**Morphotype**	**28°C**	**37°C**
rdar (curli and cellulose)	42 (60%)	10 (14.29%)
bdar (curli)	13 (18.6%)	20 (28.57%)
saw (neither curli nor cellulose)	15 (21.4%)	40 (57.14%)

Upon incubation on Congo red agar plates at 28°C, 42 (60%) isolates formed the rdar morphotype with curli and cellulose formation, 13 (18.6%) isolates produced only curli, and 15 (21.4%) isolates produced neither curli nor cellulose, showing the saw morphotype. When the bacteria were incubated at 37°C, only 10 (14.29%) isolates produced the rdar morphotype, whereas 20 (28.57%) isolates formed the bdar morphotype and 40 (57.14%) isolates produced the saw morphotype.

## 4. Discussion

In this study, a total of 70 *E. coli* isolates were identified and tested for the presence of genes linked to virulence and antibiotic resistance, as well as Class 1, 2, and 3 integrons, plasmid profiles, and biofilm formation potential. The findings underscore significant reservoirs of antimicrobial resistance in this environment and carry implications for both public and occupational health.

### 4.1. Prevalence of Antibiotic Resistance Genes

All isolates carried the *uid*A gene encoding the *β*‐glucuronidase, confirming their identity as *E. coli* [16]. Among diarrheagenic virotypes, EI*EC* was the most frequent (five isolates), followed by enterotoxigenic (ET*EC*, two isolates) and a single enterohemorrhagic strain (EH*EC*). The detection of *ipaH* (in 7.1% of isolates) confirmed EI*EC* strains, while the presence of *eltB* and *estA* in two isolates (2.86%) indicated ET*EC* carrying heat‐labile and heat‐stable toxins [17]. One isolate harbored both *vt1* and *eaeA*, suggesting an EH*EC* lineage. Notably, no isolates carried *vt2*, *bfpA*, *ial*, or *pCVD*, indicating the absence of enteroaggregative and typical enteropathogenic *E. coli* in our sample set, consistent with prior classifications of diarrheagenic *E. coli* in Southeast Asia [17]. These virulence profiles illustrate the diversity of pathogenic *E. coli* in slaughterhouse effluents, which could pose infection risks if humans or animals are exposed. Fifteen distinct ARGs were identified among the isolates, reflecting a broad multidrug resistance profile. A substantial proportion (67.1%) of the isolates harbored the *blaOXA-48* gene, a Class D carbapenemase that threatens the efficacy of critical *β*‐lactams. The *blaOXA-48* gene was first identified in carbapenem‐resistant *Klebsiella pneumoniae* in Turkey in 2001 [18]. Recent studies have reported the widespread presence of carbapenemase‐producing Enterobacteriaceae (CPE) in slaughterhouse wastewater; our findings indicate that environmental *E. coli* from slaughterhouse waste have not been exempt from this trend, highlighting the role of such facilities in the environmental dissemination of these resistant pathogens [19].

Additionally, the study detected the presence of *qnrB* and *qnrS* genes, which confer resistance to quinolones. The detection of these genes in environmental *E. coli* isolates suggests that selective pressure may occur even without direct exposure to fluoroquinolones. Furthermore, the sulfonamide resistance genes *sul1* and *sul2* were found in 4 and 14 isolates, respectively. The plasmid‐mediated *ampC* gene *blaCMY-2*, associated with reduced susceptibility to various cephalosporins and beta‐lactamase inhibitors, was also identified. These findings align with recent systematic reviews indicating the prevalence of multidrug‐resistant *E. coli* strains in slaughterhouse wastewater environments of South Asia [20]. Such MDR strains in environmental settings are concerning, as they can complicate treatment outcomes and contribute to the environmental spread of resistance [21].

### 4.2. Detection of Carbapenemase Activity

The MHT was employed to assess carbapenemase production among 11 genotypically identified *E. coli* isolates harboring *IMP-1* and *IMP-4* resistance genes. Of these, five isolates tested positive for imipenemase production and five for meropenemase production. The detection of resistance genes in isolates that tested negative in the MHT could reflect assay′s limitations in identifying specific *β*‐lactamase functions, as noted in prior studies. The detection of IMP‐type CPEs in slaughterhouse wastewater underscores the potential public health risks associated with environmental dissemination of these resistant pathogens [19].

### 4.3. Colistin Resistance Genes

The study screened for five colistin resistance genes (mcr‐1 to mcr‐5) but did not detect their presence in any isolates. Phenotypic MIC tests revealed that the highest MIC observed was 2 *μ*g/mL in five isolates, while the majority (52 isolates) exhibited an MIC90 of ≤ 0.5 *μ*g/mL. This suggests that most isolates remain susceptible to colistin. The absence of *mcr* genes in our study is somewhat reassuring and aligns with some recent environmental studies that found low or sporadic *mcr* prevalence in wastewater. For example, a 2021 report on abattoir effluents noted variable detection of *mcr-1* across different facilities, indicating that colistin resistance has not uniformly disseminated in such setting [19]. It should be noted that CLSI has updated its recommendations for colistin testing in later editions (M07, M100, and MR01, 2020), but our study followed the guidance available at the time of experimentation.

### 4.4. Presence of Class 1 Integrons

Class 1 integrons were detected in 22.86% (16/70) of the isolates, reinforcing their established association with multidrug resistance profiles in environmental and clinical strains. A significant correlation was observed between the presence of integrons and resistance genes such as *blaTEM*, *sul1*, *sul2*, and *tetA*. This suggests that integrons play a crucial role in the dissemination of resistance genes, a finding consistent with recent research highlighting the abundance of integrons harboring antimicrobial resistance gene cassettes in aquatic environments [20]. Interestingly, some genes typically linked to Class 1 integrons, for example, *dfrA12/17*, were also observed in integron‐negative isolates, suggesting that other mobile genetic elements (e.g., plasmids, IS elements, or Tn21 family transposons) may mediate their carriage. This phenomenon has been reported in related contexts; for example, Ahmed et al. [20] noted that some *E. coli* from Dhaka wastewater carried *dfrA* genes on mobile elements not identifiable as integrons [22]. Although our study did not explore the exact genetic contexts of these genes, the pattern underscores the complexity of resistance gene dissemination and signals a need for future genomic or mobilome analyses.

### 4.5. Plasmid Association With Resistance Genes

In untreated effluents from WWTPs in Dhaka, *E. coli* isolates frequently harbored plasmids encoding resistance to *β*‐lactams and fluoroquinolones, including *blaTEM* and *qnrS* genes [23], which reinforces our findings on plasmid‐borne resistance determinants in slaughterhouse isolates. The study found that isolates harboring multiple resistance genes often contained plasmids, whereas isolates without plasmids exhibited a lower prevalence of multiple ARGs. This underscores the role of plasmids in the horizontal transfer of resistance genes. Recent metagenomic analyses have revealed a diverse array of plasmid‐mediated ARGs in slaughterhouse wastewater, emphasizing the importance of monitoring and controlling plasmid dissemination to mitigate the spread of antimicrobial resistance [24].

### 4.6. Biofilm Formation and Associated Genes

The ability to form biofilms was assessed in 70 *E. coli* isolates by evaluating the presence of biofilm‐associated genes and phenotypic expression of curli and cellulose. Every isolate examined carried the *fimH* gene, which encodes Type 1 fimbriae—a structural feature recognized for its role in initiating biofilm development. The *papC* gene, associated with P fimbriae, was detected in 7.14% of isolates; however, no direct correlation was found between *papC* presence and biofilm formation in vitro. The *csgA* gene, encoding the major subunit of curli fimbriae, was also identified; but interestingly, the extent of curli or cellulose expression did not strictly predict the overall biofilm mass in a quantitative assay. These results mirror recent findings from an Algerian poultry slaughterhouse study, which reported a complex relationship between genotype and biofilm phenotype [25].

The distinction between weak and moderate biofilm producers matters because even weak producers can adhere to surfaces like slaughterhouse drains or equipment, surviving longer than planktonic cells. In our case, all *E. coli* isolates had at least some biofilm capacity (via *fimH*, curli, and cellulose), so each has potential for environmental persistence. Moderate producers, by forming more robust biofilm structures, likely resist cleaning or disinfection better and act as more persistent reservoirs of resistance. Recent work supports this: For instance, a 2025 study on *E. coli* and biocide susceptibility from a slaughterhouse environment in Nigeria noted that isolates surviving disinfection often had moderate biofilm formation, making complete removal difficult without targeted cleaning protocols [26]. Also, a broad review of wastewater biofilms (2025) shows biofilms in wastewater act as reservoirs of ARGs and enhance horizontal gene transfer [27]. Highlighting those points allows us to conclude that even modest biofilm formation in slaughterhouse wastewater is not trivial—it contributes to survival, environmental spread, and potential gene exchange.

Finally, the detection of critical resistance genes such as *bla*OXA‐48, *bla*IMP, ESBLs, and plasmid‐mediated quinolone resistance in *E. coli* from slaughterhouse wastewater indicates significant risks of exposure for both workers and surrounding communities. Similar concerns have been reported in South Asia, where livestock and poultry workers in Pakistan′s Pothohar region were found to carry multidrug‐resistant *E. coli* and *Staphylococcus aureus* due to close contact with animals and waste under poor hygiene conditions [28]. Surveys from poultry farms in Multan, Punjab, also revealed extensive misuse of last‐resort antibiotics such as colistin and enrofloxacin, intensifying selective pressure for AMR dissemination into the environment [29]. In India, recent studies of meat and food handlers similarly demonstrated high contamination rates with MDR *E. coli* and *Salmonella*, reinforcing that occupational exposure is a key transmission pathway [30]. Together, these findings suggest that in Dhaka—where wastewater treatment is often limited—slaughterhouse effluents could act as both a direct occupational hazard and a broader environmental reservoir of AMR, underscoring the urgent need for effluent treatment, protective measures for workers, and integrated One Health monitoring.

## 5. Conclusion

This study reveals a substantial burden of antimicrobial resistance and biofilm‐forming ability in *E. coli* strains recovered from slaughterhouse wastewater. Notably, none of the isolates was classified as strong biofilm formers, likely due to their wastewater origin. Bacteria in liquid effluents remain largely planktonic and thus lack the selective pressure to develop the robust biofilms seen on persistent solid surfaces. The identification of resistance determinants targeting key antibiotic classes—such as *β*‐lactams, sulfonamides, and quinolones—raises serious concerns regarding the environmental dissemination of resistant pathogens. The findings emphasize the need for comprehensive wastewater treatment strategies to mitigate the dissemination of antimicrobial resistance. In particular, critical gaps in current wastewater management practices call for the adoption of advanced treatment technologies and strengthened monitoring protocols. Integrating slaughterhouse effluents into broader AMR surveillance frameworks would allow for early detection and a more effective public health response. To better grasp the broader implications, future studies should include diverse microbial populations and expanded sampling to assess the spread and impact of resistance traits in similar wastewater environments. Our findings align with previous studies on municipal WWTPs in Dhaka [20], which also report a high prevalence of MDR *E. coli*, suggesting that urban wastewater—including slaughterhouse discharge—constitutes a significant environmental reservoir of antibiotic resistance.

## Ethics Statement

The authors have nothing to report.

## Disclosure

The funding agency had no role in the study design, data collection, data analysis, interpretation of results, or the decision to publish.

## Conflicts of Interest

The authors declare no conflicts of interest.

## Funding

This study was funded by the Ministry of Education under the GARE (LS2018785).

## Supporting information


**Supporting Information** Additional supporting information can be found online in the Supporting Information section. Table S1: All the details of primer and target gene information for PCR. Table S2: The odds ratios and confidence intervals for statistical analysis of associations between Class 1 integrons and AMR genes. Figures S1 and S2 are the images of gel electrophoresis, indicating the presence of virulence genes and plasmid DNA, respectively.

## Data Availability

All data generated or analyzed during this study is included in this published article and its supporting information.
