# Supplementary material for: Slaughterhouse Wastewater as a Reservoir of Thermotolerant E. coli With Antimicrobial Resistance and Virulence Potential in Dhaka, Bangladesh
Source: Int J Microbiol. 2025 Dec 6;2025:2875935. doi: 10.1155/ijm/2875935 (PMC12752870; doi:10.1155/ijm/2875935)
Supplement: Supplementary file 1 — Supporting Information Additional supporting information can be found online in the Supporting Information section. Table S1: All the details of primer and target gene information for PCR. Table S2: The odds ratios and confidence intervals for statistical analysis of associations between Class 1 integrons and AMR genes. Figures S1 and S2 are the images of gel electrophoresis, indicating the presence of virulence genes and plasmid DNA, respectively. [file IJM-2025-2875935-s001.docx]

Supplementary Table 1: Primer and target genes information for PCR

| **Target gene** | **Primer name** | **Primer sequence (5′-3′)** | **Amplicon size (bp)** | **Annealing T(°C)** | **References** |
| --- | --- | --- | --- | --- | --- |
| *uidA* | uidA(F)  uidA(R) | TATGGAATTTCGCCGATTTT  TGTTTGCCTCCCTGCTGCGG | 166 | 55.2 | [21] |
| *eltB* | LT-F | TCTCTATGTGCATACGGAGC | 322 | 54 |  |
|  | LT-R | CCATACTGATTGCCGCAAT |  |  |  |
| *estA* | ST-F | GCTAAACCAGTA^G^ GGTCTTC  A | 147 | 55 |  |
|  |  | AAAA |  |  | [14] |
|  | ST-R | CCCGGTACA^G^ GCAGGATTAC  A  AACA |  |  |  |
| *vt1* | vt1-F | GAAGAGTCCGTGGGATTACG | 130 | 55 |  |
|  | vt1-R | AGCGATGCAGCTATTAATAA |  |  |  |
| *vt2* | vt2-F | ACCGTTTTTCAGATTTT^G^ CA  A | 298 | 55 |  |
|  |  | CATA |  |  |  |
|  | vt2-R | TACACAGGAGCAGTTTCAGA CAGT |  |  |  |
| *eaeA* | eaeA-F | CACACGAATAAACTGACTAA | 376 | 57 |  |
|  | eaeA-R | A |  |  |  |
|  |  | ATGAAAAACGCTGACCCGC |  |  |  |
|  |  | ACCTAAAT |  |  |  |
| *ial* | Shig-F | CTGGTAGGTATGGTGAGG | 320 | 55 |  |
|  | Shig-R | CCAGGCCAACAATTATTTCC |  |  |  |

| *bfpA* | bfpA-F  bfpA-R | TTCTTGGTGCTTGCGTGTCTT TT | 367 | 55 |  |
| --- | --- | --- | --- | --- | --- |
|  |  | TTTTGTTTGTTGTATCTTTGT AA |  |  |  |
| pCVD | EA-F | CTGGCGAAAGACTGTATCAT | 630 | 55 |  |
|  | EA-R | CAATGTATAGAAATCCGCTG TT |  |  |  |
| *ipaH* | ipaH-F ipaH-R | TGGAAAAACTCAGTGCCTCT CCAGTCCGTAAATTCATTCT | 422 | 55 | [22] |
| *blaTE M* | TEM-F TEM-R | ATAAAATTCTTGAAGACGAA  GACAGTTACCAATGCTTAAT C | 971 | 50 | [23] |
| *blaCM* | CMY2-F | GCTGAGAGCTCATGATGAAA | 1146 | 54 | [24] |
| *Y 2* |  | AAATCG |  |  |  |
|  | CMY2-R | GGTACGGATCCTTATTGCAG C |  |  |  |
| *blaCT* | CTX- | CACACGTGGAATTTAGGGAC | 996 | 56 |  |
| *X-M- 15* | M15-F  CTX- | T |  |  | [25] |
|  | M15-R | GCCGTCTAAGGCGATAAACA |  |  |  |
| *blaOX* | OXA-1F | ACACAATACATATCAACTTC | 814 | 54 |  |
| *A-1*  *group* | OXA-1R | GC  AGTGTGTTTAGAATGGTGAT |  |  |  |
|  |  | C |  |  |  |
| *blaOX* | OXA-1A- | TCAACTTTCAAGATCGCA | 609 | 47 |  |
| *A-47* | F |  |  |  |  |
|  | OXA-1B- R | GTGTGTTTAGAATGGTGA |  |  |  |
| *blaND* | NDM-F | GGTTTGGCGATCTGGTTTTC | 465 | 58 |  |
| *M-1* | NDM-R | CGGAATGGCTCATCACGATC |  |  |  |

| *blaSH V* | SHV-F  SHV-R | CACTCAAGGATGTATTGTG TTAGCGTTGCCAGTGCTCG | 885 | 52 | [26] |
| --- | --- | --- | --- | --- | --- |
| *blaOX A-48* | OXA-F OXA-R | TTGGTGGCATCGATTATCGG  GAGCACTTCTTTTGTGATGG C | 438 | 54 | [27] |
| *sul1* | Sul1-F Sul1-R | GTGACGGTGTTCGGCATTCT TTTACAGGAAGGCCAACGGT | 668 | 58 | [28] |
| *sul2* | Sul2-F Sul2-R | GGCAGATGTGATCGACCTCG  ATGCCGGGATCAAGGACAA G | 405 | 58 |  |
| *qnrB* | QnrB-F  QnrB-R | GGMATHGAAATTCGCCACTG  TTTGCYGYYCGCCAGTCGAA | 264 | 55 | [29] |
| *qnrS* | QnrS-F QnrS-R | GCAAGTTCATTGAACAGGGT  TCTAAACCGTCGAGTTCGGC G | 428 | 56 |  |
| *dfrA1* | DfrA1-F  DfrA1R | ACGGATCCTGGCTGTTGGTT GGACGC  CGGAATTCACCTTCCGGCTC GATGTC | 254 | 70 | [30] |
| *dfrA17* | DfrA17-F  DfrA17- R | GTCGCCCTAAAACAAAGTTA CGCCCATAGAGTCAAATGT | 195 | 53 | [31] |
| *tetA* | TetA-F  TetA-R | GTAATTCTGAGCACTGTCGC  CTGCCTGGACAACATTGCTT | 577 | 55 |  |
| *blaKP C1* | KPC1-F KPC1-R | TGTCACTGTATCGCCGTC  CTCAGTGCTCTACAGAAAAC C | 900 | 58 | [27] |
| *blaKP C2* | KPC2-F  KPC2-R | CGGAACCTGCGGAGTGTATG | 802 | 56 |  |

|  |  | CAGCAGTTCAGGCCAACAG G |  |  |  |
| --- | --- | --- | --- | --- | --- |
| *blaKP C3* | KPC3F  KPC3R | ATGTCACTGTATCGCCGTCT  TTTTCAGAGCCTTACTGCCC | 911 | 55 |  |
| *blaVI M1* | VIM-1F VIM-1R | TTATGGAGCAGCAACCGATG T  CAAAAGTCCCGCTCCAACG A | 801 | 60 |  |
| *blaVI M2* | VIM-2F  Vim-2R | ATTGGTCTATTTGACCGCGTC  TGCTACTCAACGACTGAGCG | 748 | 56 |  |
| *blaIM P1* | IMP-1F  IMP-1R | CTACCGCAGCAGAGTCTTTG  AACCAGTTTTGCCTTACCAT | 610 | 56 |  |
| *blaIM P4* | IMP-4F IMP-4R | ATGAGCAAGTTATCTGTATTC T  AGTGTGTCCCGGGCCACC | 741 | 58 |  |
| *mcr1* | MCR-1F  MCR-1R | CTCATGATGCAGCATACTTC  CGAATGGAGTGTGCGGTG | 320 | 58 | [32] |
| *mcr2* | MCR-2F MCR-2R | TGTTGCTTGTGCCGATTGGA  AGATGGTATTGTTGGTTGCT G | 715 | 58 |  |
| *mcr3* | MCR-3F MCR-3R | AAATAAAAATTGTTCCGCTT A  TGAATGGAGATCCCCGTTTT T | 929 | 58 |  |
| *mcr4* | MCR-4F  MCR-4R | TCACTTTCATCACTGCGTTG\  TTGGTCCATGACTACCAATG | 1116 | 58 |  |
| *mcr5* | MCR-5F MCR-5R | ATGCGGTTGTCTGCATTTATC  TCATTGTGGTTGTCCTTTTCT G | 1644 | 58 |  |

| intI | intI F intI R | CCTCCCGCACGATGATC TCCACGCATCGTCAGGC | 280 | 50 | [33] |
| --- | --- | --- | --- | --- | --- |
| *fim*H | *fim*H-F *fim*H-R | TGCAGAACGGATAAGCCGTG G  GCAGTCACCTGCCCTCCGGT A | 508 | 60 | [34] |
| *pap*C | *pap*C-F *pap*C-R | GCAGTCACCTGCCCTCCGGT A  GTGGCAGTATGAGTAATGAC CGTTA | 205 | 60 |  |
| *csgA* | *csgA-F*  *csgA-R* | ACTCTGACTTGACTATTACC  AGATGCAGTCTGGTCAAC | 200 | 60 | [35] |
| *fliC* | Fli15 Typ04 | CGG TGT TGC CCA GGT TGG TAA T  ACT GGT AAA GAT GGC T | 620 | 55 | [36] |
| *papG* | *papG-F*  *papG-R* | CTGTAATTACGGAAGTGATT  ACTATCCGGCTCCGGATAAA | 1070 | 63 | [37] |

Supplementary Table 2: Statistical analysis of associations between class 1 integrons and AMR genes

| **Gene** | **Detected_plus** | **Detected_minus** | **Not_plus** | **Not_minus** | **p_value_fisher** | **odds_ratio** | **p_value_chisq** | **logP** |
| --- | --- | --- | --- | --- | --- | --- | --- | --- |
| blaTEM | 12 | 2 | 4 | 52 | 1.36338E-08 | 67.55608792 | 3.80009E-10 | 7.865384 |
| blaCTX-M-15 | 3 | 13 | 13 | 41 | 0.748286325 | 0.730976913 | 0.655998924 | 0.125932 |
| blaOXA-1 | 0 | 16 | 16 | 38 | 0.014811147 | 0 | 0.013175401 | 1.829411 |
| blaOXA-47 | 0 | 16 | 16 | 38 | 0.014811147 | 0 | 0.013175401 | 1.829411 |
| blaOXA-48 | 4 | 12 | 12 | 42 | 1 | 1.164019729 | 0.816223029 | 0 |
| blaCMY 2 | 5 | 11 | 11 | 43 | 0.49806208 | 1.761135354 | 0.36268798 | 0.302717 |
| blaMP1 | 2 | 14 | 14 | 40 | 0.329472456 | 0.412698534 | 0.261312841 | 0.482181 |
| blaMP4 | 5 | 11 | 11 | 43 | 0.49806208 | 1.761135354 | 0.36268798 | 0.302717 |
| sul1 | 16 | 0 | 0 | 54 | 4.03211E-16 | Inf | 5.93045E-17 | 15.39447 |
| sul2 | 12 | 4 | 4 | 50 | 2.37749E-07 | 33.89691192 | 1.55663E-08 | 6.623881 |
| qnrB | 5 | 15 | 11 | 39 | 0.762838712 | 1.17892802 | 0.78713651 | 0.117567 |
| qnrS | 7 | 9 | 9 | 45 | 0.039341383 | 3.79778364 | 0.023454185 | 1.40515 |
| dfrA1 | 10 | 6 | 6 | 48 | 8.92014E-05 | 12.59912039 | 1.71182E-05 | 4.049628 |
| dfra17 | 4 | 12 | 12 | 42 | 1 | 1.164019729 | 0.816223029 | 0 |
| tetA | 8 | 8 | 8 | 46 | 0.006305438 | 5.565722136 | 0.003242055 | 2.200285 |


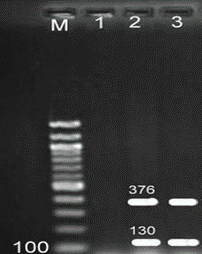


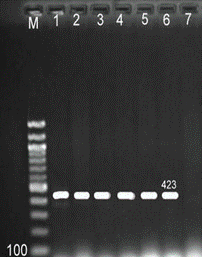

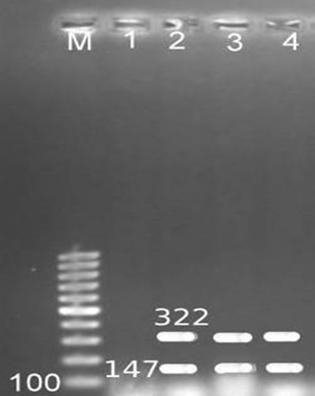


(a) (b) (c)

Supplementary Figure 1: Detection of virulence genes (a) *eltB* and *estA* for ETEC, (b) *eaeA*


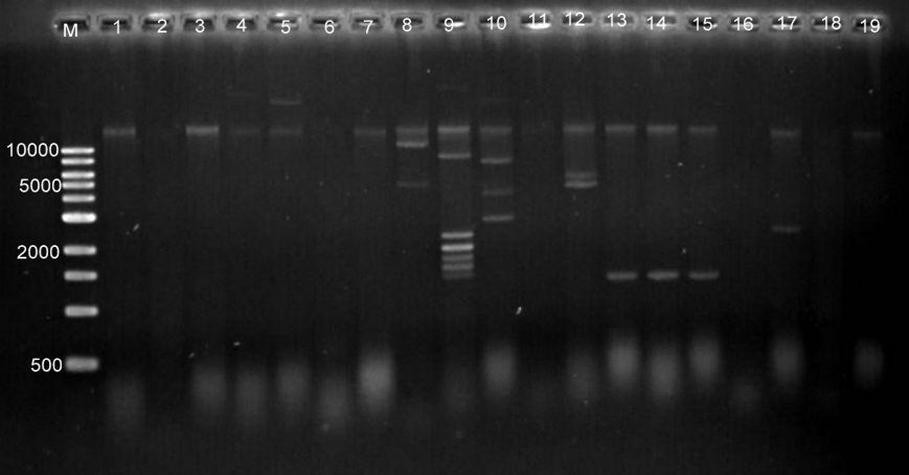
and *vt1* for EHEC, (c) *ipaH* for EIEC among isolated samples.

Supplementary Figure 2: Agarose gel (1%) electrophoresis of representative plasmid DNA
